# Rare cause of neonatal apnea from congenital central hypoventilation syndrome

**DOI:** 10.1186/s12887-022-03167-8

**Published:** 2022-02-24

**Authors:** Prakarn Tovichien, Krittin Rattananont, Narathorn Kulthamrongsri, Mongkol Chanvanichtrakool, Buranee Yangthara

**Affiliations:** 1 Division of Pulmonology, Department of Pediatrics, Faculty of Medicine Siriraj Hospital, Mahidol University, Bangkok, Thailand; 2 Faculty of Medicine Siriraj Hospital, Mahidol University, Bangkok, Thailand; 3 Division of Neurology, Department of Pediatrics, Faculty of Medicine Siriraj Hospital, Mahidol University, Bangkok, Thailand; 4 Division of Neonatology, Department of Pediatrics, Faculty of Medicine Siriraj Hospital, Mahidol University, Bangkok, Thailand

**Keywords:** Case report, cogenital central hypoventilation syndrome, CCHS, neonatal apnea, PHOX2B

## Abstract

**Background:**

Congenital central hypoventilation syndrome (CCHS) is a rare condition caused by mutations in the Paired-Like Homeobox 2B (PHOX2B) gene. It causes alveolar hypoventilation and autonomic dysregulation. This report aimed to raise awareness of this rare cause of neonatal apnea and hypoventilation as well as described the diagnostic work up to confirm the diagnosis in resource-limited setting where polysomnography for neonate is unavailable.

**Case presentation:**

A late preterm female newborn born from a non-consanguineous primigravida 31-year-old mother had desaturation soon after birth followed by apnea and bradycardia. After becoming clinically stable, she still had extubation failure from apnea without hypercapnic ventilatory response which worsened during non-rapid eye movement (NREM) sleep. After exclusion of other etiologies, we suspected congenital central hypoventilation syndrome and sent genetic testing. The result showed a PHOX2B gene mutation which confirmed the diagnosis of CCHS. We gave the patient’s caregivers multidisciplinary home respiratory care training including tracheostomy care, basic life support, and simulation training for respiratory problem solving. Then, the patient was discharged and scheduled for follow-up surveillance for associated conditions.

**Conclusion:**

Diagnosis of CCHS in neonates includes the main clue of the absence of hypercapnic ventilatory response which worsens during non-rapid eye movement (NREM) sleep after exclusion of other causes. Molecular testing for *PHOX2B* gene mutation was used to confirm the diagnosis.

## Background

Congenital central hypoventilation syndrome (CCHS) is a rare genetic disorder caused by mutations in the Paired-Like Homeobox 2B (*PHOX2B*) gene. It causes sleep-related hypoventilation and autonomic nervous system dysregulation [1, 2] which is associated with Hirschsprung disease and neural crest tumors such as neuroblastomas, ganglioneuromas and ganglioneuroblastomas [3, 4].

CCHS typically presents in the neonatal period usually shortly after birth. Presentation of CCHS in newborns were first reported in 1970 [5]. Some patients may also present in late childhood or adulthood (late-onset CCHS).

CCHS classically manifests as hypoventilation which is worsening in non-rapid eye movement (NREM) sleep, unusually it presents with hypoventilation during waking hours except for severe cases and rarely presents with apnea [6]. The main respiratory manifestation is the incapability of breathing regulation in response to abnormality of CO_2_ and O_2_ blood concentration, which is termed as hypercapnia and hypoxemia, respectively.

The most common cause for CCHS is identified as autosomal dominant inheritance in the *PHOX2B* gene [3, 4, 7]. Most *PHOX2B* gene mutations occur de novo (65–95%) [8]. Other familial *PHOX2B* gene mutations can be from somatic mutations in symptomatic parents (25%), asymptomatic parents (5–25%) with somatic mosaicism or germline mosaicism [7, 9, 10].

Most of CCHS cases (90–92%) are heterozygous for an abnormal expansion of polyalanine repeat sequence due to polyalanine repeat expansion mutations (PARM) in exon 3 of the *PHOX2B* gene, nevertheless, the rest (8–10%) are other non-polyalanine repeat expansion mutation (NPARM) in *PHOX2B* gene [3, 4, 7, 11].

Here we reported a unique case of a newborn with apnea and hypoventilation without hypercapnic ventilatory response which worsened during non-rapid eye movement (NREM) sleep, which was finally diagnosed as CCHS. We aim to raise awareness of this rare cause of neonatal apnea and hypoventilation, describe the diagnostic work up to exclude other etiologies, and confirm the diagnosis in resource-limited setting where polysomnography for neonate was unavailable.

## Case presentation

A late preterm 36-week female neonate was born from a non-consanguineous primigravida 31-year-old mother who had regular antenatal care, no underlying disease, normal diabetic screening, no risk of major thalassemia, no history of smoking, illicit drug, or alcohol use. The mother’s serology was normal for hepatitis B virus, VDRL, and anti-HIV.

During pregnancy, she developed isolated idiopathic polyhydramnios with many episodes of fetal bradycardia and had placental angioedema. The mother came up with labor pain and the membrane was artificially ruptured. No systemic analgesia was given during delivery.

The neonate was delivered vaginally, weighted 2,700 g and had an Apgar score of 6 in the 1st minute, 7 in the 5th minute, and 10 in the 10th minute. She then had bradypnea (respiratory rate 30 breaths/min) and oxygen desaturation down to 80% at 10 min of age. After putting on an oxygen hood at 10 L per min (LPM), oxygen saturation was increased up to 95–100%. At that time, her respiratory rate was 30 breaths/min. She received antibiotics because neonatal sepsis was suspected. Two hours after delivery, the neonate had an apneic episode with bradypnea and oxygen desaturation down to 49%. The patient was then put on a nasal continuous positive airway pressure (nCPAP) and transferred to the neonatal intensive care unit (NICU). Her chest radiograph showed only perihilar streaking.

During hospitalization, the patient had multiple episodes of bradycardia, apnea and underwent endotracheal intubation at the age of 3 days. Multiple investigations were performed to find the cause. A chest radiography (CXR) showed no abnormal finding. An electrocardiogram (EKG) showed sinus bradycardia which was suspected from respiratory impairment. An electroencephalogram (EEG) didn’t show electroclinical seizure. A brain MRI showed no abnormality of the brain stem. A diaphragmatic ultrasonography showed symmetrical diaphragmatic motion without paradoxical movement. The patient’s complete blood count (CBC), electrolyte and serum glucose levels were normal. The septic workups which included a hemoculture as well as a cerebrospinal fluid (CSF) analysis and culture were also normal. Thus, apnea of prematurity (AOP), though, unlikely to occur with such severity at her birth gestational age, was suspected at that time and intravenous aminophylline was initiated as a treatment.

At 5 days of age, the patient had delayed passing meconium. A physical examination showed abdominal distension. There was bile content on orogastric tube. The barium enema was performed and showed relatively small caliber and abnormal contraction of the rectum without definite transition zone. Therefore, Hirschsprung disease couldn’t be totally excluded. The patient did not undergo rectal biopsy for a definitive diagnosis at that time because she could pass stools well after rectal irrigations.

**Fig. 1:**
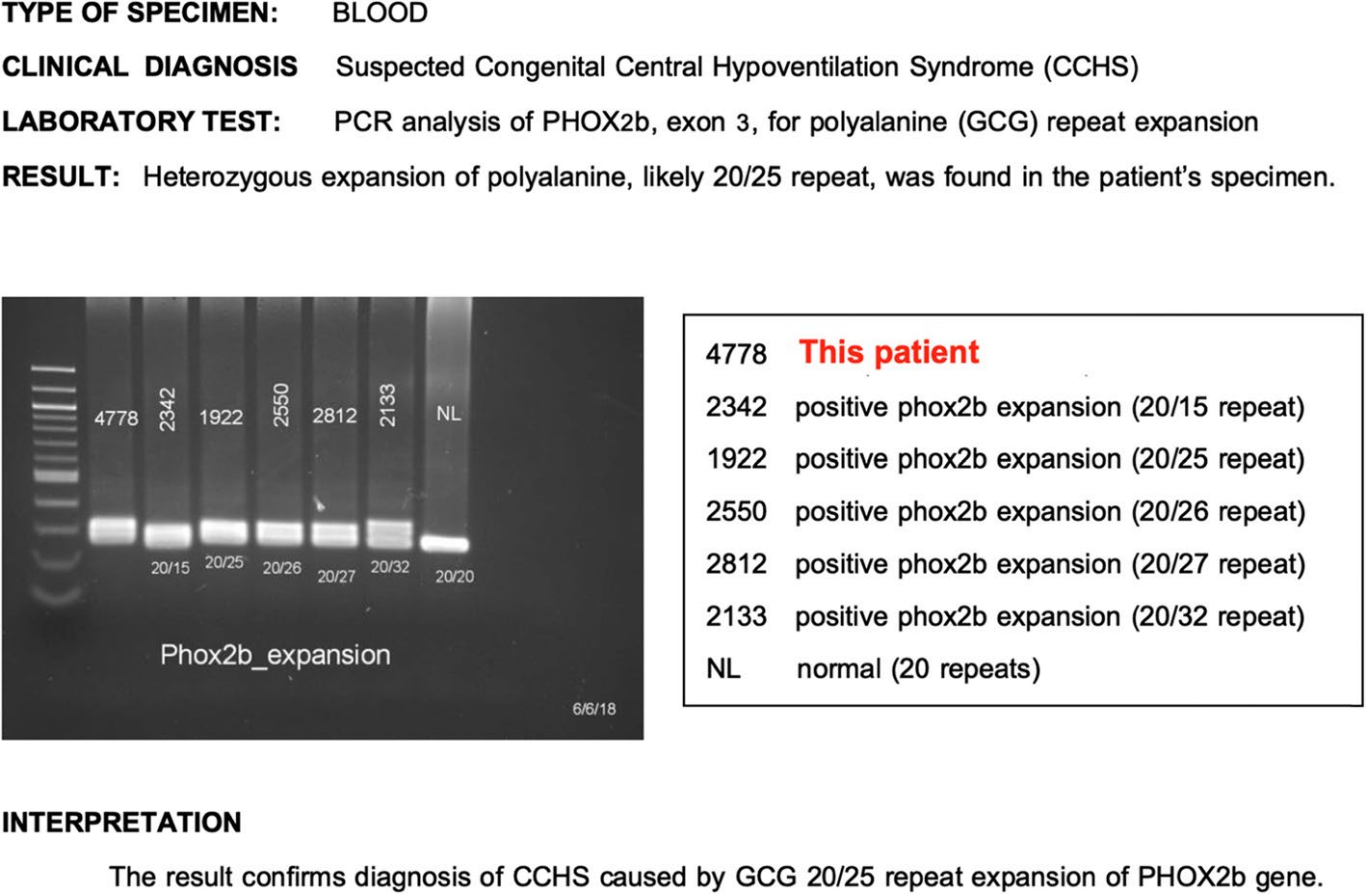
PCR analysis of PHOX2B gene, exon 3, for polyalanine (GCG) repeat expansion in this child’s specimen (4778) showed heterozygous expansion of polyalanine, likely 20/25 repeat. The samples derive from the same experiment and that gels/blots were processed in parallel

The patient could breathe with ventilator support and her respiratory rate was 40–60 breaths/min. During sleep, the respiratory rate usually dropped to 40 breaths/min without spontaneous breath. However, blood gases and end tidal CO_2_ levels were normal. The first 2 extubation attempts at the age of 18 and 37 days failed because the patient became drowsy, had stridor and desaturation. Fiberoptic laryngoscopy and bronchoscopy were performed and revealed only erythema and swelling of the arytenoid without laryngomalacia or other airway abnormalities. The results of our investigation are summarized in Table 1. The third extubation to nasal CPAP was performed at the age of 50 days (post-conceptual age of 43 weeks). Intravenous dexamethasone was administered to reduce airway swelling before the extubation. After the extubation the patient had no stridor nor other signs of respiratory distress, but her monitor tracing showed bradypnea, desaturation, and hypoventilation without hypercapnic ventilatory response to respiratory acidosis. Her capillary blood gas confirmed severe respiratory acidosis (pH 7.11, PCO_2_ 89 mmHg). We strongly suspected a diagnosis of congenital central hypoventilation syndrome and sent genetic testing of the *PHOX2B* gene mutation. The result showed heterozygous expansion of polyalanine, caused by GCG 20/25 repeat expansion (Fig. 1). Unfortunately, we couldn’t do a full attended polysomnography because small size equipment for neonates was unavailable.

**Table 1:** Summarized results of the investigations

Investigation	Result
CBC	Hb 15.9 g/dl Hct 47.1%
	WBC 16,950/uL (N70% L21% M8% E1%)
	Plt 246,000/uL
Electrolyte	Na 141, K 4.1, Cl 101, HCO_3_ 18 mEq/L
ABG	pH 7.20, pCO_2_ 76.8, pO_2_ 39.3, HCO_3_ 22.8
CXR	Normal
EKG	Sinus bradycardia
EEG	No electroclinical seizure
MRI brain	No abnormality of the brain stem
Diaphragmatic ultrasonography	Symmetrical diaphragmatic motion without paradoxical movement
Fiberoptic laryngoscope and bronchoscope	Erythema and swelling of arytenoid without laryngomalacia or other airway abnormalities
PCR analysis of *PHOX2B* gene mutation	Heterozygous expansion of polyalanine caused by GCG 20/25 repeat expansion

The patient was discharged at 3 months of age after a tracheostomy insertion. We gave multidisciplinary home respiratory care training including tracheostomy care, basic life support, simulation training for respiratory problem solving, and provided home visits. We changed the tracheostomy tube every 6 weeks and did follow-up surveillance including neurocognitive development.

At 3 years of age, the child presented with abdominal distension and a repeat barium enema was suggestive of Hirschsprung disease. She underwent trans anal anorectal pull-through surgery. A biopsy of the sigmoid colon revealed aganglionosis at proximal end and throughout the entire length of specimen confirming Hirschsprung disease.

Until now, the child was well managed on home ventilator support. Although the parents took good care of her, she had two episodes of pneumonia. She gradually caught up with her growth and development. Her mother can perform home respiratory care proficiently and we hope that she can be decannulated in the future.

## Discussion and conclusion

Congenital central hypoventilation syndrome (CCHS) is a rare condition, first reported in 1970 [5]. It may be underdiagnosed. At that time, there were about 20 case reports of idiopathic central hypoventilation syndrome in adults. In 1999, The American Thoracic Society indicated there were roughly 160–180 cases of CCHS around the world [12]. Until 2003, the *PHOX2B* gene mutations, located on chromosome 4q12, were considered responsible for CCHS leading to diagnosis of more cases [4]. In 2005, the French CCHS working group reported an estimated incidence of 1: 200,000 live births in France [13]. In 2009, about 1,000 cases were confirmed by molecular studies [14]. Since then, more than 1000 cases of CCHS have been reported worldwide [8].

The clinical presentation of CCHS is variable from shallow breathing during sleep, neonatal cyanosis, central apnea during wakefulness to sudden death [15, 16]. In our patient, the first manifestation was neonatal apnea and bradycardia. The diagnosis of CCHS in preterm infants (gestational age < 37 weeks) could be challenging and significantly delayed, especially in early or extremely premature infants (gestational age < 28 weeks), because they commonly have AOP which has similar presentation as that of CCHS. Unlike CCHS, apnea in AOP frequently occurred in rapid eye movement (REM) sleep and almost all infants eventually outgrow AOP at post-conceptual age of 36 to 40 weeks. However, in extremely premature infants AOP could exist beyond 38 to 40 weeks [17]. Without the aid of polysomnography, the diagnosis of CCHS was suspected even with its rarity in our patient because apnea and bradypnea still persist at post-conceptual age of 43 weeks and other causes including cardiopulmonary, neuromuscular, and metabolic causes had been ruled out. The unusually severe and frequent apnea requiring endotracheal intubation in late preterm infants could be another important clue to the diagnosis of CCHS.

During ventilator support, our patient still had hypoventilation during non-rapid eye movement (NREM) sleep. After other causes had been ruled out, the diagnosis of CCHS was suspected even with the rareness of the disease.

The diagnosis of CCHS was supported by polysomnography and confirmed by molecular testing for *PHOX2B* gene mutation, the disease defining gene [4, 7]. Her genetic testing results was heterozygous of polyalanine expansion 20/25. The polyalanine repeat mutation (PARM) is found in over 90% of CCHS. The genotype of PARM coding ranges from 20/24 to 20/33 (The normal genotype is 20/20). The 20/25 genotype, as in our patient, is one of the most common mutations of *PHOX2B* gene [8, 14].

The respiratory severity of CCHS is related to the number of repeats in PARM. Her genotype mutation is 20/25 which has the mildest form of respiratory symptom. A patient with this type of mutation has a variety of symptom severities. Most patients do not require 24 h of ventilatory support while they grow up [8]. Moreover, the PARM length is also associated with several autonomic dysregulation-related symptoms such as arrhythmia, abnormal sweating and GI symptoms [7]. In our patient, she had only bradycardia and constipation as the autonomic symptom.

Constipation in CCHS patients could be from autonomic dysfunction or Hirschsprung disease [3]. Hirschsprung disease occurs among 20% of CCHS patients. It was reported in 87 to 100% of NPARMs in contrast to 13 to 20% of PARMs. There was no report in 20/25 genotype before. At 3 years of age, this child was finally diagnosed Hirschsprung disease and was the first case in 20/25 genotype to the best of our knowledge.

All CCHS patients should be evaluated for 3 main issues which are breathing physiology during asleep and awake, autonomic nervous system function, and comorbidities screening. All evaluations and screening processes will be individualized to each patient who has different forms of mutations on the *PHOX2B* gene [8]. For 20/25 mutations as in this patient, the guideline suggests annual in-hospital comprehensive physiologic testing, neurocognitive assessment, and 72 h Holter monitoring.

There are many ways of assisting ventilation such as invasive ventilation, noninvasive positive pressure ventilation, and diaphragmatic pacing. Invasive ventilation with a tracheostomy tube is suitable for the infant period as in the case of this patient. Noninvasive positive pressure ventilation is usually used in school-aged patients during sleep. Diaphragmatic pacing can also be considered in some severe cases who depend on mechanical ventilator during daytime but it is costly and needs to be performed by experienced tertiary care centers [18].

Early diagnosis and multidisciplinary management can help when predicting disease outcomes and long-term survival. Two-thirds to 80% of patients are able to breathe on their own during waking hours after infancy while the rest need to use a mechanical ventilator all the time. Effective ventilation support can reduce mortality [19]. While there are all kinds of complications, severe pneumonia is the most common cause of death with CCHS patients. Continuing abnormalities in breathing require lifelong ventilatory support [20]. Neurologic complications including learning disabilities and attention deficit hyperactivity disorder may result from affected brain development as a consequence of chronic hypoxia [21]. Although CCHS is a lifelong disorder and there are many life-threatening complications, many CCHS patients eventually have a good quality of life and normal social functioning.

In conclusion, the absence of a hypercapnic ventilatory response which worsens during non-rapid eye movement (NREM) sleep, after exclusion of other causes, is the main clue to the diagnosis of CCHS in neonates. Molecular testing for *PHOX2B* gene mutation was used to confirm the diagnosis.

## Data Availability

This data is available from the authors upon reasonable request and with the permission of the institution.

## Abbreviations

CCHS: Congenital central hypoventilation syndrome
PHOX2B: Paired-Like Homeobox 2B
NREM: Non-rapid eye movement
PARM: Polyalanine repeat expansion mutation
NPARM: Non-polyalanine repeat expansion mutation
LPM: Liter per min
nCPAP: Nasal continuous positive airway pressure
NICU: Neonatal intensive care unit
